# Investigation and confirmation of differentially expressed miRNAs, as well as target gene prediction in papillary thyroid cancer, with a special emphasis on the autophagy signaling pathway

**DOI:** 10.22099/mbrc.2022.43844.1751

**Published:** 2022

**Authors:** Mojtaba Zehtabi, Zahra Akbarpour, Sepehr Valizadeh, Yousef Roosta, Amir Mahdi Khamaneh, Mortaza Raeisi

**Affiliations:** 1 Hematology and Oncology Research Center, Tabriz University of Medical Sciences, Tabriz, Iran; 2 Rahat Breathe and Sleep Research Center, Tabriz University of Medical Sciences, Tabriz, Iran; 3 Department of Internal Medicine, School of Medicine, Tabriz University of Medical Sciences, Tabriz, Iran; 4 Department of Internal Medicine, School of Medicine, Imam Khomeini Hospital, Urmia University of Medical Sciences; 5 Department of Molecular Medicine, Faculty of Advanced Medical Sciences, Tabriz University of Medical Sciences, Tabriz, Iran

**Keywords:** Papillary thyroid carcinoma, Autophagy, mRNA, miRNA

## Abstract

Papillary thyroid carcinoma (PTC) is the most common endocrine cancer. However, the role of biomechanics in the development and progression of PTC is obscure. The microarray dataset GSE104005 was examined to identify important microRNAs (miRNAs or miRs) and their probable roles in the carcinogenesis of PTC. The gene expression omnibus (GEO) database was used to obtain the data. R was used to access the differentially expressed miRNAs (DEMs) and genes (DEGs). The multiMiR software was used to predict DEM targets. To validate the top DEMs and DEGs, thirty tissue samples were obtained from PTC patients who had their thyroids removed and compared with 30 normal samples. The total RNA content of the tumor and corresponding non-tumoral adjacent samples were purified and converted to cDNA. Expression levels of top dysregulated miRNAs and their target and predicted DEG were evaluated using the RT-qPCR method. miR-182 and miR-183 were top upregulated miRs and miR-30d was the most downregulated miR among DEMs. Furthermore, FOXO1 which was shown to be targeted by aforementioned miRNAs, was the most downregulated genes among other DEGs. 10 hub nodes were detected by PPI construction. PTEN was the hub node with highest score. The i*n vitro *gene expression analysis was also showed the same expression pattern in tissues. Significant increase in miR-182-5p and miR-183-5p expressions, as well as a significant decrease in FOXO1 and miR-30d-5p expressions, suggest that PTC cells may tend to preserve their autophagy capability.

## INTRODUCTION

Thyroid gland is an endocrine organ with a remarkable risk of cancer. Thyroid cancer has become more common in recent decades [1]. Almost all thyroid cancers develop in the follicular epithelium, which can be classified into three types: papillary thyroid carcinoma (PTC), follicular thyroid carcinoma (FTC), and anaplastic thyroid carcinoma (ATC), with PTC being the most common and is responsible for about 80% of all thyroid malignancies [2-4]. The high recurrence rate explains why the burden of this malignancy is likewise high in the Iranian population [5, 6]. As a result, additional knowledge about the pathophysiology of PTC is critical. The importance of miRNAs in cancer research has gotten a lot of attention in recent years. The expression features of miRNA are strongly associated to the development, progression, and prognosis of many malignancies, making it a significant element in carcinogenesis and metastasis [7, 8]. Previous researches has identified certain key miRNAs that interfere with cancer development based on miRNA expression patterns [9, 10]. Bioinformatics analysis was also frequently employed for the discovery of new biomarkers and mechanism research. In this study, we aimed to find and validate hub miRNAs that play essential roles in the formation and survival of PTC, so that we could learn more about the mechanisms and therapeutic applications of PTC. The focus of this study was to confirm miRNAs which involve in the autophagy pathway.

## MATERIALS AND METHODS

**Data collection and preprocessing: **The microRNA expression profile GSE104005 was downloaded from the Gene Expression Omnibus (GEO) database (https://www.ncbi.nlm.nih. gov/geo/). Quantile normalization was performed to normalize the dataset. This dataset, based on Illumina HumanHT-12 WG-DASL V4.0 R2 expression beadchip, was made up 29 thyroid carcinoma samples and 5 non-neoplastic thyroids. In this case, we included 20 PTC samples as our inclusion criteria and compare the miRNA expression profiles with control samples. 

**Identification of DE-miRNAs and DEGs: **R software, version 4.1.1, was used to analyze the data. Quantile normalization approach was used to normalize the data in this case. The Gplot software is also used to create boxplots using normalized data. To detect DE-miRNAs and DEGs, the Limma (version 3.483), data.table (version 1.14), and GEOquery (version 2.60) packages were downloaded and utilized. In our analysis, p< 0.05 and |Log2FC| > 1 were used as cutoff criterion. Finally, the Pheatmap (version 1.0.12) and Enhancedvolcano (version 1.0.10) packages were used to display the heatmap and volcano diagram, respectively. 

**Identification of target genes: **Once we have a list of possible miRNAs, the next step is to determine the target genes of these miRNAs so that we may run further analyses based on them, such as gene ontology. Multimir package which predicts target genes from mirTarbase and Tarbase databases, was used to anticipate target genes and in common genes between two databases were elucidated and illustrated by Venndiagram package. The procedures taken result in the identification of a large number of genes that may be utilized to discover molecular and cellular mechanisms in the progression of PTC. 

**Functional Enrichment Analysis of Putative Target Genes: **To supplement Gene Ontology (GO) and KEGG pathway analyses, identified target genes were uploaded to Funrich 3.1.3 software p 0.05 was used as the inclusion criterion for both GO and KEGG. GO is a popular method for linking genes, gene products, and sequences to biological phenomena. KEGG is a database that allows scientists to analyze genomic sequences and other high-throughput biological data.

**PPI network construction: **Following functional enrichment analysis, a protein-protein interaction network was constructed using STRING and Cytoscape software. The target gene was selected from the STRING database with a score of >0.9, and the interaction network was visualized using Cytoscape software. The CytoHubba app was also used to identify hub genes. The hub genes are a small number of genes with a large number of interaction partners that serve as an important node in the PPI network for gene interaction. By overlaying the top 10 genes, CytoHubba discovered hub genes using 11 categorization algorithms.

**Sample collection: **The demographic information of patients is summarized in Table 1. Patients with definite FNA-approved diagnoses of PTC were included. The exclusion criteria were a history of goiter or other thyroid diseases, previous chemotherapy and/or radioactive iodine therapy, and history of other simultaneous malignancies. The local institutional review board (IRB) authorized this study (approval No.IR.TBZMED.REC.1398.1262). Between March 2017 and July 2018, samples were collected at Tabriz's Imam Reza Hospital. Samples were obtained by surgical intervention from PTC patients undergoing thyroidectomy. Thirty samples of PTC tissues and thirty samples of non-cancerous neighboring tissues from the same patient were chosen. H&E staining and light microscopy were used to validate the PTC and non-cancerous tissue samples. The number of males and females was almost equal. The majority of the patients (50%) had a tumor confined to the right thyroid lobe. The majority of tumors (56.6%) were over 2 cm in size and also most of the tumors (86.7%) had not invaded the thyroid capsule.

**Table 1:** Demographic status of the patients

**Parameters**	**Items**	**Frequency**	**Percent**
**Gender / min and max age**	Female / 43 and 81	16	53.3
	Male / 49 and 73	14	46.7
**Involved Lobe**	Right	15	50.0
	Left	11	36.6
	Both	4	13.4
**Tumor/Nodule** **size**	≤ 2 cm	17	56.6
	> 2 cm	13	43.4
**Capsular** **Involvement**	Yes	4	13.3
	No	26	86.7

**RNA Extraction, cDNA synthesis, and reverse transcription-quantitative (RT-q) PCR: **Total RNA was extracted from the 300 mg purified human PTC samples using Trizol reagent (Ambion life technologies, UK) according to the manufacturer's protocol. Nanodrop device (Spectrophotometer 2000/2000c) was used to assess the quantity of purified RNA (ng/ul) and any protein or mineral residual contaminations. Complementary DNA synthesis was performed using AnaCell cDNA synthesis kit (Lot No:cs0025) following the manufacturer's temperature protocol: 70°C for 5 min, 37°C for 60min, and 70°C for 5 min. Biomir high sensitivity microRNA kit (Zistroyesh Co/Iran) provided stem-loop technology for complementary DNA synthesis for each target and housekeeping miRNA (U6). PCR, using miR-specific primers and FOXO1 primer was performed using SYBER Green PCR kit (AnaCell/Iran). Stem-loop RT amplification primers for miRNAs were provided by the kit (Zistroyesh Co/Iran) according to the manufacturer's instruction. The PCR conditions were as follows: 95°C pre-denaturation for 15 min, followed by 40 cycles of denaturation at 95°C for 20 seconds, an annealing-extension step at 60°C for 60 seconds, followed by a melt analysis step between 55°C and 95°C. The housekeeping gene to normalize the FOXO1 and miRNAs was the GAPDH (glyceraldehyde-3-phosphate dehydrogenase) and U6 gene, respectively. All RT-qPCR assays were complemented triplicate. Primer details for FOXO1 and GAPDH housekeeping gene are tabled in Table 2. 

**Table 2:** Primers for RT-qPCR

**Gene**	**Primer Sequence**	**Length (bp)**	**TM (°C)**	**GC (%)**	**Position**	**Product Length**
F (*FOXO1*)	CAAGTCATGTGGAAAGCCCAA	21	59.9	47.6	Exon 3	155
R (*FOXO1*)	ACCAAGCCAATGAAGATGCAA	21	59.5	43	Exon 3	
F (*GAPDH*)	TCTGACTTCAACAGCGACACC	21	60	52	Exon 7	117
R (*GAPDH*)	GTTGCTGTAGCCAAATTCGTT	21	60	43	Exon 8	

**Statistical Analysis: **Gene expression analysis was performed using ΔΔCt method, where ΔΔCt = ΔCt (Ct _target_ _gene_ – ΔCt _ref._ _housekeeping_ _gene_; *treated sample*) - (Ct _target_ _gene_ – Ct _ref._ _gene_; *untreated control*) [11]. To compare target and reference gene expression levels, Ct values were utilized, and fold changes reflected relative quantification. The Shapiro-Wilk test was used to check the data for normality. A paired t-test was used because the data distribution of FOXO1, miR-182-5p, miR-183-5p, and miR-30d-5p was normal. A p-value of less than 0.05 was deemed significant. Statistical analyzes and boxplot creations were performed using GraphPad Prism software version 8.0.2. Finally, the significance of correlations between the fold changes (FCs) of FOXO1 and miRNA levels were calculated using Pearson's r.

## RESULTS

As shown in Table 3, a total of 25 miRNAs were identified as differentially expressed miRNAs, 13 of which were considerably upregulated (LogFC>1) and 12 of which were significantly downregulated (LogFC<-1). To determine target genes of discovered DE-miRNAs, the MiRSystem online base and the R Multimir package were utilized. Figure S1 (in spllementary file) shows that 245 in common target genes were found between discovered target genes and DEGs for revealed DE-miRNAs. Ultimately, FOXO1 was found to be the most downregulated gene among others. 

**Table 3:** Up and down regulated miRNAas

**Symbol**	**logFC**	**P.Value**	**adj.P.Val**
MIR30D	-2.80452	0.012469	0.139065
MIR1324	-2.67584	0.000325	0.01685
MIR1255B1	-2.1366	0.00028	0.015493
MIR1322	-1.95308	0.000149	0.010275
MIR767	-1.92962	4.62E-07	0.000194
MIR764	-1.66035	3.03E-07	0.000146
MIR921	-1.6075	1.48E-05	0.002169
MIR548A1	-1.40674	0.002184	0.052751
MIR197	-1.40671	0.00057	0.023769
MIR636	-1.36666	4.54E-06	0.000913
MIR548B	-1.25039	3.23E-05	0.003615
MIR2116	-1.13933	0.021667	0.18754
MIR199A2	1.083402	0.027498	0.21286
MIR382	1.084819	0.005249	0.086496
MIR892B	1.096889	4.47E-05	0.00448
MIR548G	1.1027	0.015898	0.158567
MIR602	1.11852	0.006074	0.093766
MIR140	1.161334	0.007288	0.10337
MIR26B	1.164595	0.003218	0.066711
MIR423	1.341283	4.44E-06	0.000901
MIR579	1.540776	0.018785	0.174137
MIR941-2	1.85384	0.000503	0.021818
MIR657	2.390512	8.00E-05	0.006868
MIR183	2.487364	0.043252	0.215338
MIR182	2.534713	0.046603	0.280545

**Note:** logFC: log fold change; adi.P.val: adjusted p value

On the aforementioned possible target genes, GO functional and KEGG pathway enrichment analysis were conducted. The following tables shows the enriched GO functions for the target genes, which include transcription regulatory activity in the (MF) category (Table 4), nucleoside, nucleotide, and nucleic acid metabolism in the biological processes (BP) category (Table 5), and nucleus in addition to cytoplasm in the cellular component (CC) category (Table 6). Furthermore, the enriched KEGG pathways for target genes of dysregulated miRNAs identified six signaling pathways, including validated targets of C-MYC transcriptional repression, proteoglycan syndecan-mediated signaling events, LKB1 signaling pathways, PAR1-mediated thrombin signaling events, and thrombin/protease-activated receptor (PAR) pathway at the top of the list (Table 7).

**Table 4:** Enriched molecular functions from GO analysis of DEGs

**Molecular function**	**No. of genes **	**Percentage of genes**	**P-value**
Transcription regulator activity	23	9.8712446	0.000472
Transcription factor activity	23	9.8712446	0.000556
Kinase binding	3	1.2875536	0.001038
Protein serine/threonine kinase activity	10	4.2918455	0.005726
Ubiquitin-specific protease activity	11	4.72103	0.009814
Chromatin binding	2	0.8583691	0.011691
Phosphatase regulator activity	2	0.8583691	0.017537
DNA-directed DNA polymerase activity	2	0.8583691	0.02685
Kinase regulator activity	2	0.8583691	0.034867
Protein translocase activity	1	0.4291845	0.038074

**Table 5:** Enriched biological processes from GO analysis of DEGs

**Biological process**	**No. of genes **	**Percentage of genes**	**P-value**
Regulation of nucleobase, nucleoside, nucleotide and nucleic acid metabolism	62	26.609442	1.02E-05
Regulation of cell proliferation	3	1.2875536	0.003072
Regulation of gene expression, epigenetic	4	1.7167382	0.0103
Immune cell migration	1	0.4291845	0.012856
Regulation of cell cycle	3	1.2875536	0.036935
Morphogenesis	1	0.4291845	0.038076
Cytoskeletal anchoring	1	0.4291845	0.050444
Chromosome segregation	1	0.4291845	0.086608
Carbohydrate metabolism	1	0.4291845	0.098355
Transcription	2	0.8583691	0.118017

**Table 6:** Enriched Cellular components from GO analysis of DEGs

**Cellular component**	**No. of genes **	**Percentage of genes**	**P-value**
Nucleus	130	68.062827	4.36E-15
Cytoplasm	114	59.685864	5.4E-09
Cyclin-dependent protein kinase holoenzyme complex	3	1.5706806	7.49E-05
Centrosome	21	10.994764	0.000146
Ruffle	4	2.0942408	0.000249
Spindle pole	4	2.0942408	0.000249
Nucleoplasm	16	8.3769634	0.000288
Integral to nuclear inner membrane	2	1.0471204	0.00051
Outer kinetochore of condensed nuclear chromosome	2	1.0471204	0.00051
Kinetochore	6	3.1413613	0.00118

**Table 7:** Enriched Biological pathways from GO analysis of DEGs

**Biological pathway**	**No. of genes **	**Percentage of genes**	**P-value**
Validated targets of C-MYC transcriptional repression	11	9.7345133	1.08E-08
Proteoglycan syndecan-mediated signaling events	50	44.247788	3.1E-08
LKB1 signaling events	49	43.362832	3.67E-08
PAR1-mediated thrombin signaling events	48	42.477876	8.79E-08
Thrombin/protease-activated receptor (PAR) pathway	48	42.477876	9.02E-08
Endothelins	48	42.477876	1.07E-07
ErbB receptor signaling network	48	42.477876	1.19E-07
Sphingosine 1-phosphate (S1P) pathway	48	42.477876	1.19E-07
TRAIL signaling pathway	48	42.477876	1.8E-07
Insulin Pathway	47	41.59292	1.97E-07

**Figure 1:**
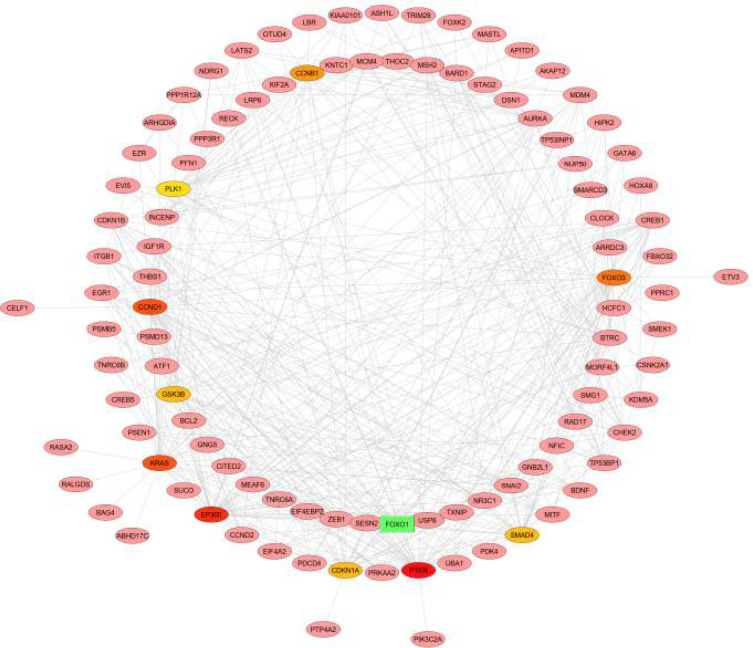
Protein-protein interaction network of enriched genes

In addition to determining the activities of protein products derived from the analyzed genes, the interaction of these products was investigated, revealing a huge network of molecular linkages between proteins. As shown in the Figure 1 the STRING database revealed that several of the target genes interacted with one another. The top ten hub nodes with the highest scores were selected for better visibility. PTEN, EP300, KRAS, CCND1, FOXO3, CCNB1, CDKN1A, GSK3B, SMAD4, and PLK1 were identified as hub genes. PTEN has the highest node score (49) of these genes.

By analysis of FOXO1 mRNA levels in the PTC and adjacent normal tissues, a 2.66-fold decrease in the expression levels of FOXO1 was observed in the PTC samples in comparison with adjacent normal tissues. This outcome was statistically significant (p<0.0001). When miR-182-5p levels were compared to non-cancerous neighboring samples, there was a substantial significant 1.73-fold increase in the expression level of this miRNA in the PTC (p=0.007). The examination of miR-183-5p expression levels in PTC and normal surrounding tissues, revealed that this miRNA was expressed significantly 2.35-fold higher in PTC samples (p=0.0001).

By comparing the expression levels of miR-30d-5p in PTC and neighboring normal tissues, we discovered a 2.62-fold significant decrease in the expression of this miRNA in PTC samples compared to adjacent normal tissues (p=0.0156 Overall results are depicted *in *Table 8. Eventually, none of the miRNA fold changes were significantly correlated with the FOXO1 fold change in the PTC and non-cancerous tissues.

**Table 8:** Analysis of FOXO1 mRNA and miR-182-5p, miR-183-5p and miR-30d-5p alterations in expression in PTC tissues in comparison to normal adjacent (as control (depicted as Control)

**mRNA / miRNA**	**Sample Status**	**Mean ± SD**	**Fold change**	**p-value**
FOXO1	PTC	-0.3767 ± 1.36	2.66	< 0.0001
	Control	1.418 ± 1.373		
miR-182-5p	PTC	-9.136 ± 1.201	1.73	0.007
	Control	-10.14 ± 1.414		
miR-183-5p	PTC	-9.075 ± 0.9741	2.35	< 0.0001
	Control	-10.67 ± 1.556		
miR-30d-5p	PTC	-4.668 ± 3.989	2.62	0.0156
	Control	-2.128 ± 1.906		

## DISCUSSION

A bioinformatics method was employed in this work to discover potential treatment targets and pathways in the PTC. Following the research, 25 DEMs were discovered, comprising 13 upregulated DEMs and 12 downregulated DEMs. Hsa-mir-182 and hsa-mir-183 were the most upregulated genes, whereas hsa-mir-30d was the most downregulated, indicating that they may act as carcinogens or tumor suppressors in PTC. Furthermore, FOXO1 was the most downregulated gene among DEGs, and the above-mentioned miRNAs were anticipated to target it bioinformatically. C-MYC transcriptional repression, proteoglycan syndecan-mediated signaling events, LKB1 signaling pathways, PAR1-mediated thrombin signaling events, and the thrombin/protease-activated receptor (PAR) pathway were significantly enriched in target genes of dysregulated DEMs, according to Go and KEGG analysis results. The involvement of hsa-mir-182 [12, 13], hsa-mir-183 [14, 15], hsa-mir-30d [16, 17], and FOXO1 [18, 19] in the autophagy signaling pathway have been explored in previous studies. Autophagy has been researched in a number of diseases, including cancer [20, 21]. Despite the inactivation of autophagy-related genes in malignancies, these genes are still active in thyroid cancer [22]. Cancerous cells may benefit from this lysosomal recycling process [23]. FOXO1 is a tumor suppressor gene, due to its function as a transcription factor of genes located in anti-neoplastic pathways including autophagy [18, 19]. In parallel, acetylated FOXO1 activates autophagy in a manner independent of the transcriptional activity of this protein [18]. As mentioned earlier, despite the tumor suppressor role of autophagy, it can be recruited by cancer cells to overcome stressful tumor microenvironment [24, 25].

In an address to transcription factor-independent activity of FOXO1, our findings indicate a 2.66-fold (p<0.0001) significant decrease in mRNA level of FOXO1 in PTC compared to normal adjacent (control) tissues suggesting an overall insufficiency in the related tumor suppressor pathways. This reduction can be explained by our results, including the miR-182-5p and miR-183-5p significant increase in expressions of 1.73- (p=0.007) and 2.35-fold (p<0.0001), respectively. The correlations of these alterations in tumor tissues are not statistically significant and this finding can be attributed to the small sample sizes (30 for both PTC and non-cancerous tissues). However, using the DIANA-TarBase v8 experimentally supported miRNA targets database, the prediction score of these two miRNAs suppressing FOXO1 is estimated as 0.821 and 0.783 out of 1 for miR-182-5p and miR-183-5p, respectively. Therefore, upregulation of these miRNAs, may indicate the epigenetic regulation of the FOXO pathway. According to this inhibitory effect of these two miRNAs on the FOXO1 tumor suppressor gene and by considering their positive effect on tumor progression, this data can be used to design future studies to bring up the repression of miR-182-5p and miR-183-5p as a part of PTC treatment methods [26, 27]. 

Interestingly, previous studies demonstrate that the ATG5 gene plays an important role in establishing autophagy [28]. This gene is targeted and downregulated by miR-183-5p in cancerous conditions [29]. Due to our results about significant 2.35-fold (p<0.0001) upregulation of this miRNA in the PTC samples, this fact brings up the hypothesis that the autophagy machinery may be affected in an additional way in the PTC and it supports our conjecture about the significant compensatory decrease in expression level of miR-30d-5p in the PTC samples aiming to preserve the autophagy capability. It is also indicated that the autophagy activity is increased in the PTC by upregulation of either autophagy-related genes or autophagy-inducing long non-coding RNAs (lncRNAs) [30]. This statement also verifies our estimation about the tendency of PTC cells to keep their autophagy machinery active.

Despite our hypothesis about the beneficial impact of autophagy in PTC, recent investigations indicate an inverse relationship between autophagy and PTC progression. For instance, it is demonstrated that miR-524-5p enhances autophagy by targeting FOXO1 mRNA resulting in the PTC development suppression [31]. It is also depicted that miR-221 and miR-222 repress the ATG10 (an essential gene in the autophagy pathway) in the PTC and these autophagy-deficient cells display more aggressiveness [32]. In addition, one study done on a PTC cell line demonstrated that downregulation of lncRNA RP11–476D10.1 along with miR-138-5p upregulation promotes autophagy and inhibits PTC cell proliferation [33]. 

Collectively, our findings of a significant decrease in the expression levels of FOXO1 and miR-30d-5p in the PTC compared to normal adjacent tissues suggest that the PTC cells may tend to preserve their autophagy activity by considering the role of autophagy in the cancer cell homeostasis. These data were gained by bioinformatic tools and validated in vitro. Therefore, this concept in parallel to other studies of autophagy may be utilized as potential items for future PTC research. Furthermore, our data can be used by future investigations.

## Acknowledgements:

This research is financially supported by Tabriz Hematology and Oncology Research Center.

## Conflict of Interest:

The authors have stated that they have no possible conflicts of interest in respect to this publication to disclose.

## Supplementary Materials


