# Supplementary figures and images for: Investigation and confirmation of differentially expressed miRNAs, as well as target gene prediction in papillary thyroid cancer, with a special emphasis on the autophagy signaling pathway
Source: Mol Biol Res Commun. 2022;11(4):173–81. doi: 10.22099/mbrc.2022.43844.1751 (PMC9905749; doi:10.22099/mbrc.2022.43844.1751)

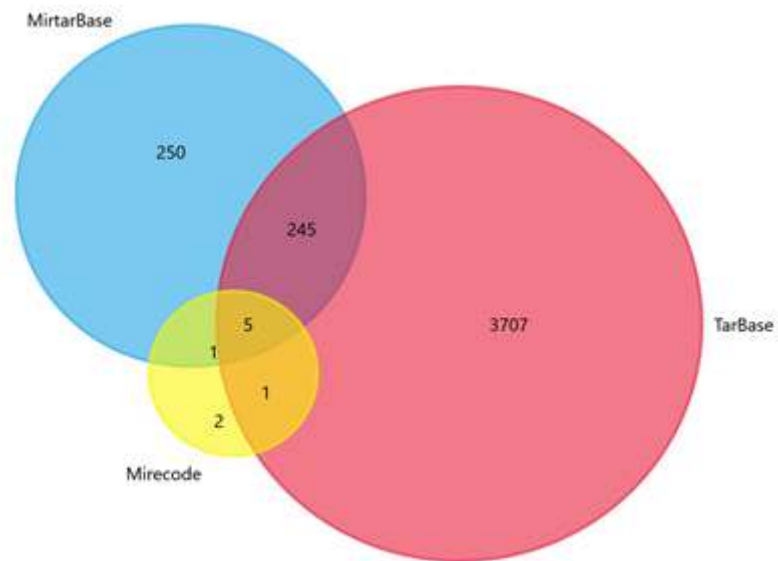

**Figure S1:** Venn diagram shows that 245 genes were overlapped between 3 different databases

Supplement: Supplementary file 1 [file mbrc-11-173-s1.pdf]
